# Analysis of metabolites of red seabream (*Pagrus major*) from different geographical origins by capillary electrophoresis time-of-flight mass spectrometry

**DOI:** 10.1371/journal.pone.0270280

**Published:** 2022-07-21

**Authors:** Junho Yang, Jiyoung Shin, Eunji Cha, Hyunsuk Kim, Yoonhyeung Lee, Soi Kim, Iseul Choi, Jiyoung Yang

**Affiliations:** 1 Department of Food Science & Technology, Pukung National University, Busan, South Korea; 2 Institute of Food Science, Pukyung National University, Busan, South Korea; Fisheries and Oceans Canada, CANADA

## Abstract

Red seabream (*Pagrus major*), a migratory fish, is characterized by high protein levels in the muscle. South Korean and Japanese red seabreams have a general distribution pattern; however, distinguishing them based on their geographical origin is difficult. In this study, we used capillary electrophoresis time-of-flight mass spectrometry (CE-TOF/MS) to analyze the red seabream muscle metabolome to investigate how can distinguish the origin of the fish. The metabolites were extracted using 50% acetonitrile in water. Chromatographic separation was successfully used to classify the metabolite profiles of Japanese and South Korean red seabream. Principal component analysis and hierarchical cluster analysis showed good ability to categorize the samples according to their origin. Amino acids showed the greatest quantitative difference in South Korean and Japanese muscle samples. Specifically, the L-alanine, L-glutamic acid, L-isoleucine, dimethylglycine, and L-valine levels in Japanese red seabream samples were significantly higher than those in South Korean samples. In contrast, the levels of trimethylamine N-oxide and inosine monophosphate in South Korean muscle samples were significantly higher than those in Japanese red muscle samples. The monitored metabolite profiles suggest that South Korean and Japanese red seabreams can be identified on the basis of amino acid levels.

## Introduction

Red seabream belongs to the family Sparidae; its appearance is characterized by red coloring on the back and tail along with small blue spots. Red seabream is rich in essential amino acids, thereby representing a valuable source of aquatic protein [1], so that it is widely consumed in South Korea, Japan, and China, particularly in the former two countries owing to the greater development of their aquaculture technologies. Recently, the Japanese Government proposed the use of ocean water to treat wastewater from the Fukushima Nuclear Power Plant. Among the radionuclides in the wastewater, including cesium 134, cesium 137, and strontium 90, tritium has raised the greatest concern [2], considering the potential exposure of humans to the element through seafood consumption. Exposure to tritium may increase the risk of cancer and mutagenesis in humans [3, 4], which inevitably raises safety concerns over seafood consumption. In addition, there have been cases of misrepresentation of the origins of aquatic products. Therefore, it is important to identify the origins of sea bream between South Korea and Japan. An earlier study elucidated the origins of olive founder (*Paralichthys olivaceus*) using gene polymorphism analyses [5], and near-infrared reflectance spectroscopy has been used to identify the origin of sea bass (*Dicentrarchus labrax* L.) [6]. However, identification of red seabream according to geographic origin using conventional molecular techniques has been challenging.

Metabolomics is the study of identification and quantification of small molecules or chemicals that can be found in cells, organs, and organisms [7]. The small molecules can include a variety of endogenous and exogenous chemicals such as peptides, amino acids, nucleic acids, carbohydrates, organic acids, minerals, and chemicals that can be ingested or synthesized in a cell or an organism as a metabolic response. Metabolomics has become an important tool in food science, including food composition analysis, food quality assessment, and food consumption monitoring. In particular, food component analysis consists of various steps such as the determination of individual protein, fat, carbohydrate, dietary fiber, and ash levels; however, metabolite analysis enables to collect more chemically detailed information [8]. Metabolomic analysis has been successfully applied to identify sea cucumber (*Apostichopus japonicus*) from different geographical locations [9]. Studies have identified regional differences among Bokbunja, Wagyu, and Holstein beef according to their distinct metabolite profiles [10, 11].

Numerous metabolomic technologies are currently available, including capillary electrophoresis (CE), nuclear magnetic resonance, gas chromatography, and liquid chromatography mass spectrometry, each of which have their advantages and disadvantages [12]. CE can be used to analyze low- and high-molecular weight polar and non-polar compounds, and time-of-flight mass spectrometry (TOF-MS) is widely used with CE owing to its advantages of speed, wide mass range, sensitivity, and high ionization transfer efficiency [13]. In particular, CE-TOF coupling combines the advantages of efficiency and low sample consumption of CE and high accuracy of TOF, along with an infinite mass range and high mass resolution. Therefore, it has particular strengths in the analysis of ionic and polar substances; CE-TOF/MS is considered to be a superior technique in biomolecular analysis [14].

Therefore, in this study, we used CE-TOF/MS to monitor the metabolomics profiles of South Korean and Japanese red seabreams to determine the origin of red sea breams and confirm the possibility of discrimination.

## Materials and methods

### Sample preparation

Japanese (n = 10) and South Korean (n = 10) red seabream were used in this study; sample information is presented in Table 1. All red seabream samples were collected at Jagalchi seafood market in Busan, as fresh edible fish. The aquaculture origin of each red seabream is presented in Table 1. This study was carried out in strict accordance with the guidelines on animal experimental ethics of the Korean Animal and Plant Quarantine Agency [15]. If a part of a dead body is collected, it does not have to be deliberated by the Ethics Committee. Therefore, this experiment did not require deliberation by the Ethics Committee. All muscle samples were harvested between the dorsal and pectoral fins on the sideline and were then stored at −80°C for the subsequent metabolite analysis. The muscle tissue samples (approximately 30 mg) were collected and mixed with 750 μl of 50% acetonitrile in water (v/v), containing methionine sulfone and camphor-10-sulfonic acid as internal standards (20 μM), and homogenized using a homogenizer (3,500 rpm, 60 s × 5 times), followed by the addition of 50% acetonitrile in water (v/v). The supernatant (400 μL) was then filtered through a 5-kDa cut-off filter (ULTRAFREE-MC-PLHCC; Human Metabolome Technologies, Yamagata, Japan) to remove macromolecules. The filtrate was centrifugally concentrated and resuspended in 50 μL of ultrapure water immediately before measurement.

**Table 1:** Basic and geographic information of red seabream samples used in this study.

Area	Coordinates (N, E)	Aquaculture depth (m)	Age (years)	Body length (cm)	Body weight (kg)
**South Korea**	34°44′19″N 128°23′47″E	10	4	45.0 ± 1.2	1.3 ± 0.3
**Japan**	34°04′08″N 136°13′54″E	10–12	4	46.7 ± 1.1	1.4 ± 0.2

### Instrumentation condition

Separations were performed using a fused silica capillary column (50 μm × 80 cm) filled with cation buffer solution to detect cations and anion buffer solutions to detect anions. The extracted metabolites were measured using an Agilent CE-TOF/MS system (Agilent Technologies Inc., Santa Clara, CA, USA) at Human Metabolome Technologies in two modes for cation and anionic metabolites [16]. The operating conditions of CE-TOF/MS for the analysis of metabolites are presented in Table 2.

**Table 2:** CE-TOF/MS conditions for obtaining the metabolome profiles from red seabream muscle samples.

Device	Agilent CE-TOFMS system (Agilent Technologies Inc.)	
**Column**	Fused silica capillary i.d. 50 μm × 80 cm	
**Condition**	Cationic Metabolite	Anionic Metabolite
**Run buffer**	Cation Buffer Solution (p/n: H3301-1001)	Anion Buffer Solution (p/n: I3302-1023)
**Rinse buffer**	Cation Buffer Solution (p/n: H3301-1001)	Anion Buffer Solution (p/n: I3302-1023)
**Sample injection**	Pressure injection 50 mbar, 10 sec	Pressure injection 50 mbar, 22 sec
**CE voltage**	Positive, 30 kV	Positive, 30 kV
**MS ionization**	ESI Positive	ESI Negative
**MS capillary voltage**	4,000 V	3,500 V
**MS scan range**	m/z 50–1,000	m/z 50–1,000
**Sheath liquid**	HMT Sheath Liquid (p/n: H3301-1020)	HMT Sheath Liquid (p/n: H3301-1020)

### Data processing and statistical analysis

Peaks detected in CE-TOFMS analyses were extracted using automatic integration software (MasterHands ver. 2.17.1.11 developed at Keio University) to obtain peak information including m/z, migration time (MT), and peak area. Detected metabolites were based on the Human Metabolome Technologies (HMT) standard library (HMT, Yamagata, Japan). The peak area was converted to relative peak area based on the metabolite peak area, internal standard peak area, and sample amount. The peak detection limit was determined based on signal-noise ratio; S/N = 3. Putative metabolites were then assigned with the HMT standard and Known-Unknown peak libraries based on m/z and MT. The tolerance was ±0.5 min for MT and ±10 ppm for m/z. If several peaks were assigned to the same candidate, the candidate was given the branch number.Principal component analysis (PCA) and hierarchical cluster analysis were performed using SampleStat ver. 3.14 (Human Metabolome Technologies Inc., Tsuruoka, Japan). Differences between metabolites in South Korean and Japanese red seabream muscle samples were analyzed using Welch’s *t*-test.

## Results and discussion

### PCA of red seabreams from different regions

**Fig 1:**
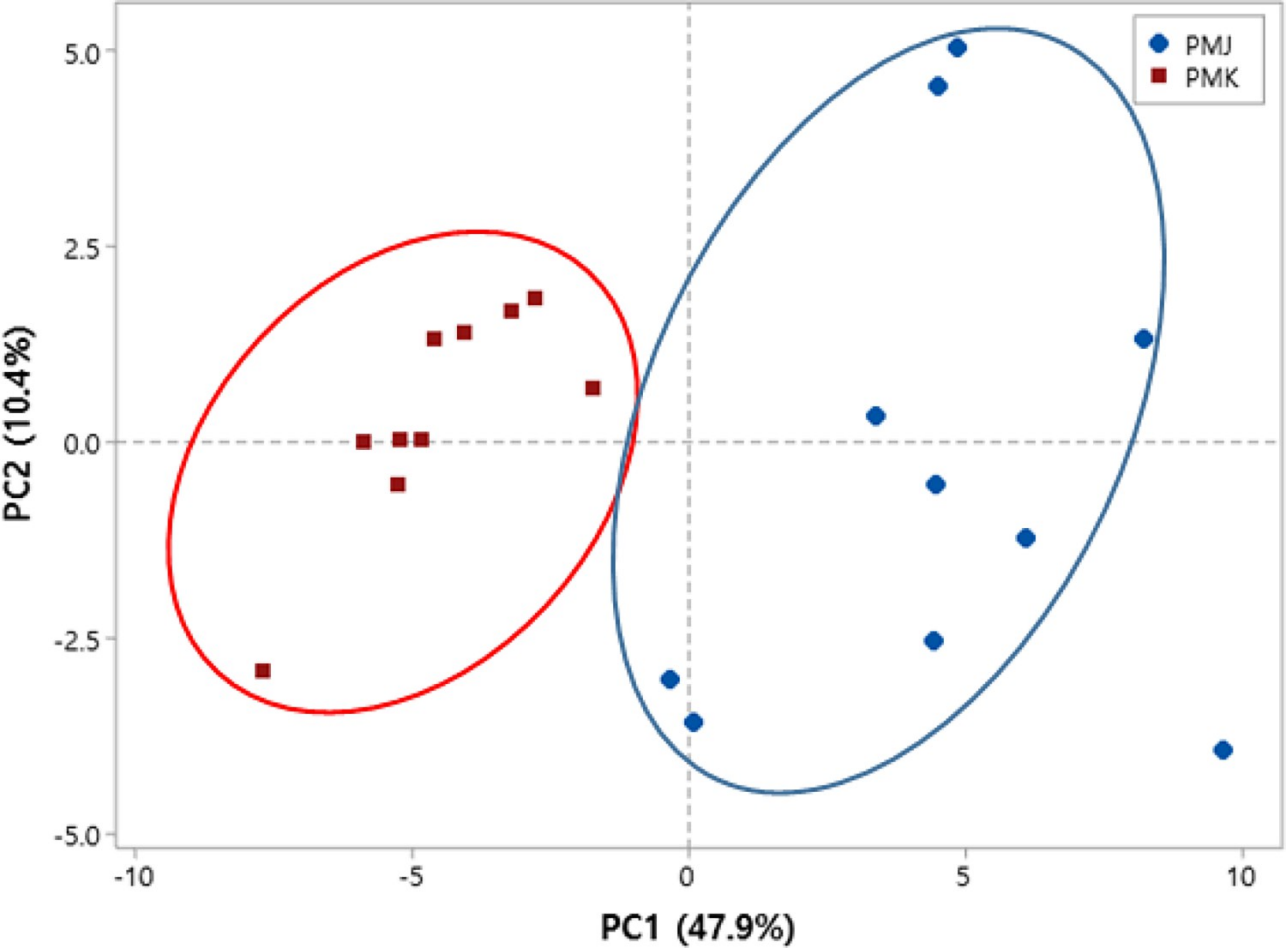
Principal component analysis of metabolome data from Japanese (PMJ, n = 10) and South Korean (PMK, n = 10) red seabream muscle samples. PMJ: Japanese red seabream (n = 10).

CE-TOF/MS presented 233 putative peaks (94 amino acids; 28 fatty acids, lipid metabolites; 17 carbohydrate metabolites; 25 nucleoside metabolites; 21 organic acids; 19 organoheterocyclic compounds; 19 organic nitrogen compounds; 10 unknown) in South Korean and Japanese red seabream muscle samples—171 in the cation mode and 62 in the anion mode (S1 Table). The principal component analysis (PCA) showed that distinct metabolic profiles were related to regional variations in South Korean and Japanese red seabream (Fig 1), with the metabolite phenotype represented in component 1 accounting for 47.9% of the total variation. The PCA score plot showed distinct metabolite phenotypes associated with different regions of red seabream. The results could provide a clear standard for distinguishing Korean red seabream from Japanese red seabream by increasing the number of samples. Additional analysis based on season and size will also be carried out.

### Differences in primary metabolites

**Fig 2:**
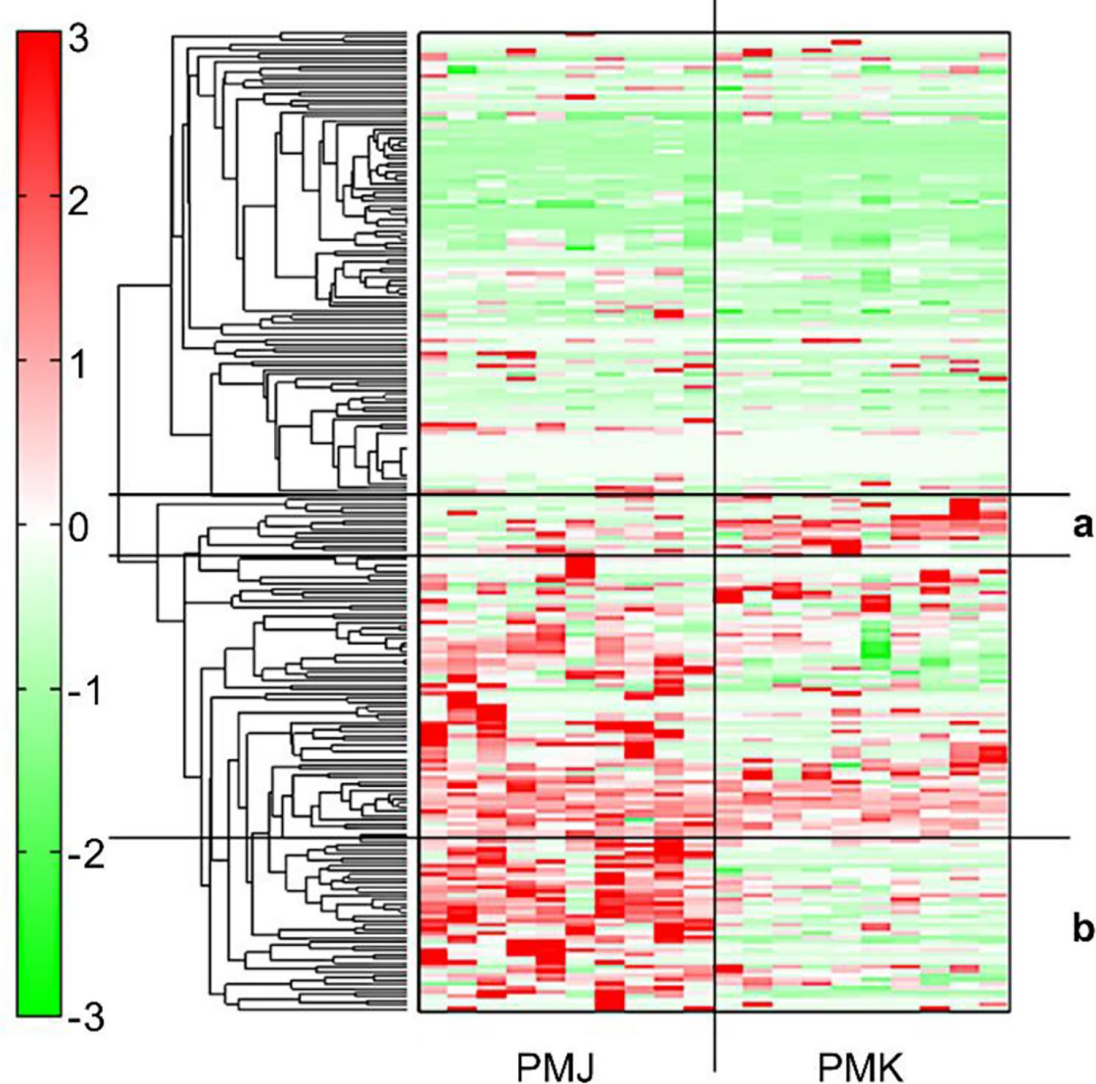
Heatmap of metabolome data from Japanese and South Korean red seabream muscle samples. The horizontal and vertical axes represent the sample names and peaks, respectively. Hierarchical clustering analysis was performed on the peaks. The distance between peaks is displayed in tree diagrams. Among the 233 metabolites, upregulated metabolites are indicated in red and downregulated metabolites are indicated in green. South Korean red seabream muscle samples (PMK, n = 10) are represented in cluster a, and Japanese red seabream muscle samples (PMJ, n = 10) are represented in cluster b.

The heatmap of the metabolome also confirmed apparent differences between South Korean and Japanese red seabream (Fig 2). The metabolites formed two clusters, with South Korean red seabream characterized by cluster a, which mainly consisted of nucleoside metabolites and organic nitrogen compounds. In contrast, Japanese red seabream was characterized by cluster b, which mainly consisted of amino acids and amino compounds. Table 3 shows the relative quantities of metabolites in South Korean and Japanese red seabream samples.

**Table 3:** Differences in the main compounds between South Korean and Japanese red seabream samples.

Compound Name	Relative area				Comparative analysis	
	PMJ		PMK		PMJ/PMK	
	Mean	SD	Mean	SD	Ratio ^1)^	*p*-value ^2)^
**l-Alanine ^c^**	1.1^−1^	1.9^−2^	7.8^−2^	2.6^−2^	1.5	0.003 **
**l-Glutamic acid ^c^**	5.0^−2^	1.2^−2^	2.9^−2^	8.5^−3^	1.7	2.7^−4^ ***
**l-Isoleucine ^c^**	7.0^−2^	1.1^−2^	4.9^−2^	1.4^−2^	1.4	0.002 **
**Dimethylglycine ^c^**	4.5^−4^	1.6^−4^	9.0^−5^	2.9^−5^	5.0	4.4^−5^***
**l-Valine ^c^**	6.6^−2^	9.4^−3^	4.9^−2^	1.2^−2^	1.3	0.003 **
**Inosine monophosphate ^a^**	1.8^−1^	2.6^−2^	2.1^−1^	1.3^−2^	0.8	0.002 **
**Trimethylamine N-oxide ^c^**	1.1^−2^	3.5^−3^	2.0^−2^	1.8^−3^	0.6	8.7^−6^ ***

Values are expressed as mean ± SD and metabolite compound (PMJ/PMK) ratio.

^1^The ratio was computed using the average detection values. The values of the PMK samples were used as the denominators.

^2^The p-value was obtained using Welch’s *t*-test. (*p < 0.05, **p < 0.01, ***p < 0.001).

^a^ = anionic mode

^c^ = cationic mode.

The levels of trimethylamine N-oxide (TMAO) and inosine monophosphate (IMP) in South Korean red seabream were significantly higher than those in Japanese red seabream. The differences in the TMAO levels can be attributed to the different distribution environments of the South Korean and Japanese red seabreams. Red seabream aquaculture depth is similar between South Korea and Japan (Table 2). However, the Japanese red seabreams were loaded onto a live fish transportation vehicle (in Japan) and shipped to South Korea. The shipping period was approximately 5 days. We believe that the live fish transport period leads to the differences in the TMAO level between South Korean and Japanese seabreams because the TMAO level varies according to the depth of the live fish transportation vehicle and the depth of the fish-culture systems. Specifically, TMAO prevents water pressure from distorting proteins and thus killing animals. Therefore, TMAO levels are generally higher in fish that live in deep waters than in those inhabiting shallow waters [17]. A previous study reported that TMAO levels tended to increase according to the depth of the water inhabited by the sea snail (*Elassodiscus tremebundus*) [18].

5’-Ribonucleotide is associated with umami taste along with glutamic acid, and IMP, guanosine monophosphate (GMP), and adenosine monophosphate (AMP) participate in 5’-ribonucleotide synthesis. In particular, IMP is abundant in meat and fish, whereas GMP is more related to plants. 5-AMP, which has a lower taste intensity than IMP or GMP, is another important umami-related substance that is widely distributed in natural food [19–21]. A regional study on loach (*Misgurnus mizolepis*) [22] and a study on wild and cultured sweetfish (*Plecoglossus altivelis*) [23] also showed differences in the IMP content, which varied with the aquaculture region. The results of the present study also showed differences in IMP levels according to region. The difference between glutamic acid and IMP levels contributes to the difference in the umami taste of red seabreams obtained from South Korean and Japanese aquaculture; therefore, this taste difference is also likely associated with the different metabolites produced in South Korea and Japanese red seabreams depending on their feed composition.

In contrast, the levels of l-Alanine, l-glutamic acid, l-isoleucine, dimethylglycine, and l-valine in Japanese red seabream samples were significantly higher than those in South Korean red seabream samples. Japanese red seabream samples were grouped into the same cluster (cluster b), which was primarily composed of amino acids and amino compounds (l-alanine, l-glutamic acid, l-isoleucine, dimethylglycine, and l-valine). However, previous studies have shown no differences in amino acid composition between cultured red seabream [1] and Israeli carp [24]. Nevertheless, this study showed relative quantity differences in amino acids in red seabream from different regions according to metabolite profiles determined by CE-TOF/MS. Amino acid analysis is used to analyze amino acid content in protein and peptide bonds in a sample. For this purpose, the peptide bond is hydrolyzed using HCL to separate the amino acids composed in the peptide bond [25]. Metabolite analysis is used to analyze all the small molecule metabolites in cells, and it differs from amino acid analysis that has been used in previous studies to determine the origin of aquatic organisms. Specifically, unlike amino acid analysis, which is used to analyze separated amino acids, metabolomic analysis using CE-MS offers the possibility to separate and identify small molecule metabolites in cells. This difference might be related to the differences between the analytical methods, amino acid analysis and metabolite analysis.

Alanine is synthesized by metabolic processes in the human body and is produced by reductive amination of pyruvic acid, and similar to threonine, serine, and glycine, it is also known to be related to saccarinity [26]. Furthermore, glutamic acid is abundant in nature, and is present in fish, seafood, pork, and beef. Glutamic acid is associated with the umami taste, which is completely distinct from the four basic tastes—sweet, salty, sour, and bitter [18, 27]. Additionally, South Korean and Japanese red seabreams showed different alanine and glutamic acid contents. This difference could possibly be attributed to the differences between their aquaculture feeds. In South Korean aquaculture, 91% melt pellets (MP) and 9% extrusion pellets (EP) are supplied to the red seabreams, whereas in Japanese aquaculture, dry pellets (DP) and EP are mainly used [28, 29]. Sand eels (*Ammodytes personatus*) are used as MP in South Korean aquaculture [30].

In contrast, the protein and lipid levels in the compound feed used in Japanese aquaculture is 55% and 10%, respectively [31]. A previous study showed significant differences in the levels of glutamic acid and alanine (also related to the umami taste) in flounder fed with MP and EP; the alanine level was higher in flounder fed EP (6.64%) than in flounder fed MP (6.05%). Similarly, glutamic acid content was higher in flounder fed EP (15.81%) than flounder fed MP (15.42%) [32].

Isoleucine is an essential amino acid for all fish species and is primarily deposited in the skeletal muscle proteins. In a study on the loss of meat quality due to dietary deficiency and excess isoleucine in grass carp (*Ctenopharyngodon idella*), differences in the shear force of grass carp muscle were associated with differences in isoleucine levels in the feed. The pH and shear force of their muscle were improved by increasing the isoleucine levels in the feed (3.8–18.5 g/kg diet). In addition, the isoleucine levels in grass carp muscle samples (3.25%–3.50%) can be increased by increasing isoleucine level in diet [33].

Valine (Val), similar to isoleucine, is a branched-chain amino acid essential for inhibiting protein synthesis and degradation in fish. Valine deficiency reportedly resulted in reduced growth performance in Mrigal carp (*Cirrhinus mrigala*) [34]. In a study on the dietary valine requirements of juvenile red seabreams, growth performance was associated with the dietary valine levels [35]. Another study on the dietary valine requirements of catla (*Catla catla*) reported the suitable dietary valine level for improved catla growth performance (1.12–1.71% valine in 0.5 1). Furthermore, catla fed a diet containing the optimal valine level show increased high-grade protein contents, as well as valine retention and gain [36]. Therefore, the current valine supply in optimized mixed feed of red sea bream seems to be a factor that causes differences in growth rate and valine content between South Korean and Japanese red sea bream.

N,N-Dimethylglycine (DMG) acts as an antioxidant metabolite via the methylation of free radicals, and as a source of glycine for glutathione synthesis. It can improve the body’s antioxidant capacity, as well as improve an animal’s ability to protect against oxidative stress and improve health [37]. Increased DMG levels in rainbow trout exposed to clove oil have been shown to be protective stress responses to anesthetics [38]. Other studies have suggested that dietary DMG could improve athletic performance in horses and mice [37, 39]. To demonstrate that Japanese red sea bream has high DMG levels, additional data is required to determine whether it is added in the diet to improve athletic performance or it is produced in response to external stress generated during the distribution process.

In the present study, the analyzed amino acids tended to be more abundant in EP-fed Japanese red seabream than in EP-fed South Korean seabream. Therefore, the differences in amino acids are considered to be due to differences in the compositions of feed supplied by South Korean and Japanese aquacultures, which is consistent with the results of a study on the effect of feed composition on the fat and amino acid composition of red seabream [40].

## Conclusion

Using metabolomics and statistical analysis, in this study, we showed a difference in the metabolite profiles between South Korean and Japanese seabream, especially with respect to amino acids (l-alanine, l-glutamic acid, l-isoleucine, dimethylglycine, and l-valine), nucleoside metabolites (IMP), and organic nitrogen compounds (TMAO). These distinct profiles are considered to be due to the difference in the aquaculture feed, which are formulated to improve fish’s conditions and the environmental factors associated with South Korean and Japanese aquaculture, respectively.

This study demonstrated the ability of metabolite profile analysis to help identify the geographic origin of red seabream by monitoring. Therefore, further research should focus on identifying specific biomarkers that can accurately determine the origin of red seabream through additional verification. These results will be used as basic research data for discriminant studies to identify the geographical origin of the red sea bream.

## Supporting information

S1 Table 233 putative metabolites (94 amino acids; 28 fatty acids, lipid metabolites; 17 carbohydrate metabolites; 25 nucleoside metabolites; 21 organic acids; 19 organoheterocyclic compounds; 19 organic nitrogen compounds; ten unknown) detected from South Korean and Japanese red seabream muscle tissue using capillary electrophoresis time of flight mass spectrometer (CE-TOF-MS) analysis. ID consists of analysis mode and number. ’C’ and ’A’ showed cation and anion modes, respectively. N.D. (Not Detected): The metabolite was below the detection limits. N.A. (Not Available): The calculation was not possible. ^¶^ In the ratio calculation, the latter was denominator. ^||^ The p-value in Welch’s t-test. * < 0.05, ** < 0.01, *** < 0.001. They were sorted by the ratio of treatment to control in descending order.
(DOCX)
