# Supplementary material for: Analysis of metabolites of red seabream (Pagrus major) from different geographical origins by capillary electrophoresis time-of-flight mass spectrometry
Source: PLoS One. 2022 Jul 21;17(7):e0270280. doi: 10.1371/journal.pone.0270280 (PMC9302829; doi:10.1371/journal.pone.0270280)
Supplement: S1 Table — ID consists of analysis mode and number. ’C’ and ’A’ showed cation and anion modes, respectively. N.D. (Not Detected): The metabolite was below the detection limits. N.A. (Not Available): The calculation was not possible. ¶ In the ratio calculation, the latter was denominator. || The p-value in Welch’s t-test. * < 0.05, ** < 0.01, *** < 0.001. They were sorted by the ratio of treatment to control in descending order. (DOCX) [file pone.0270280.s001.docx]

**Support information**

S1 Table. 233 putative metabolites (94 amino acids; 28 fatty acids, lipid metabolites; 17 carbohydrate metabolites; 25 nucleoside metabolites; 21 organic acids; 19 organoheterocyclic compounds; 19 organic nitrogen compounds; ten unknown) detected from South Korean and Japanese red seabream muscle tissue using capillary electrophoresis time of flight mass spectrometer (CE-TOF-MS) analysis

| Comparative Analysis | | | | | | | | | | |
| --- | --- | --- | --- | --- | --- | --- | --- | --- | --- | --- |
|  |  |  |  | PMJ | | PMK | | PMJ vs PMK | | |
| ID | Compound name | *m/z* | MT/RT | Mean | S.D. | Mean | S.D. | Ratio ^¶^ | *p*-value ^\|\|^ | |
| C_0055 | 1-Aminocyclopentanecarboxylic acid | 130.087 | 9.39 | N.A. | N.A. | 9.9E-05 | N.A. | <1 | N.A. |  |
| C_0016 | 1-Aminocyclopropane-1-carboxylic acid | 102.055 | 6.81 | N.A. | N.A. | N.A. | N.A. | N.A. | N.A. |  |
| C_0100 | 1-Methylhistidine 3-Methylhistidine | 170.094 | 7.27 | 1.1E-03 | 3.2E-04 | 8.9E-04 | 1.8E-04 | 1.2 | 0.108 |  |
| C_0117 | 11-Aminoundecanoic acid | 202.181 | 9.43 | 1.1E-04 | 1.9E-05 | 1.1E-04 | 2.7E-05 | 1.0 | 0.901 |  |
| C_0152 | 2'-Deoxyguanosine | 268.105 | 11.35 | 7.3E-05 | 1.2E-05 | 5.0E-05 | 1.1E-05 | 1.5 | 1.8E-04 | *** |
| C_0130 | 2'-Deoxyuridine | 229.080 | 22.90 | N.A. | N.A. | N.A. | N.A. | N.A. | N.A. |  |
| C_0115 | 2,6-Diaminopimelic acid | 191.101 | 8.72 | N.A. | N.A. | N.A. | N.A. | N.A. | N.A. |  |
| C_0043 | 2-Amino-2-(hydroxymethyl)-1,3-propanediol | 122.082 | 8.07 | 1.4E-04 | N.A. | 6.1E-05 | 3.2E-05 | 2.3 | N.A. |  |
| C_0022 | 2-Aminoisobutyric acid 2-Aminobutyric acid | 104.071 | 9.44 | 5.4E-03 | 1.4E-03 | 3.1E-03 | 5.1E-04 | 1.7 | 4.4E-04 | *** |
| A_0006 | 2-Hydroxybutyric acid | 103.040 | 9.70 | 3.6E-04 | N.A. | N.A. | N.A. | 1< | N.A. |  |
| A_0020 | 2-Hydroxyglutaric acid | 147.030 | 16.51 | 6.5E-04 | 2.7E-04 | 4.9E-04 | 1.2E-04 | 1.3 | 0.198 |  |
| C_0036 | 2-Methylserine | 120.066 | 10.19 | 2.6E-04 | 1.3E-04 | 1.2E-04 | 2.2E-05 | 2.1 | 0.007 | ** |
| A_0019 | 2-Oxoglutaric acid | 145.014 | 21.07 | N.A. | N.A. | 2.3E-03 | 4.6E-04 | <1 | N.A. |  |
| A_0009 | 2-Oxoisovaleric acid | 115.041 | 10.11 | 9.2E-04 | N.A. | N.A. | N.A. | 1< | N.A. |  |
| C_0044 | 2-Phenylethylamine | 122.097 | 7.61 | 1.6E-04 | N.A. | 1.6E-04 | 9.8E-05 | 1.0 | N.A. |  |
| C_0019 | 3-Aminobutyric acid | 104.071 | 7.75 | 4.5E-05 | N.A. | N.A. | N.A. | 1< | N.A. |  |
| C_0021 | 3-Aminoisobutyric acid | 104.071 | 7.64 | 5.9E-04 | 2.3E-04 | 3.7E-04 | 1.1E-04 | 1.6 | 0.017 | * |
| C_0049 | 3-Hydroxy-2-methyl-4-pyrone | 127.038 | 22.95 | N.A. | N.A. | N.A. | N.A. | N.A. | N.A. |  |
| A_0007 | 3-Hydroxybutyric acid | 103.040 | 9.52 | N.A. | N.A. | 7.0E-04 | N.A. | <1 | N.A. |  |
| C_0087 | 3-Methyladenine | 150.078 | 7.72 | 4.0E-05 | 2.3E-06 | 4.0E-05 | 7.2E-06 | 1.0 | 0.958 |  |
| A_0021 | 3-Phenylpropionic acid | 149.061 | 8.73 | N.A. | N.A. | N.A. | N.A. | N.A. | N.A. |  |
| C_0035 | 4-Amino-3-hydroxybutyric acid | 120.066 | 7.90 | 7.4E-05 | 4.8E-05 | 3.7E-05 | 4.4E-06 | 2.0 | 0.471 |  |
| A_0013 | 4-Methyl-2-oxovaleric acid 3-Methyl-2-oxovaleric acid 2-Oxohexanoic acid | 129.055 | 9.53 | 1.6E-03 | 2.0E-04 | 6.7E-04 | 6.4E-05 | 2.3 | 3.0E-05 | *** |
| C_0009 | 4-Methylpyrazole | 83.061 | 6.84 | N.A. | N.A. | N.A. | N.A. | N.A. | N.A. |  |
| C_0053 | 4-Oxopyrrolidine-2-carboxylic acid | 130.050 | 10.78 | 4.1E-04 | 8.5E-05 | 2.6E-04 | 6.6E-05 | 1.6 | 4.1E-04 | *** |
| C_0165 | 5'-Deoxy-5'-methylthioadenosine | 298.096 | 9.86 | 2.3E-05 | 6.5E-06 | 2.2E-05 | N.A. | 1.0 | N.A. |  |
| C_0032 | 5-Aminovaleric acid | 118.087 | 7.84 | 1.6E-03 | 1.5E-03 | 1.5E-03 | 3.3E-04 | 1.1 | 0.807 |  |
| C_0096 | 5-Hydroxylysine | 163.109 | 6.77 | N.A. | N.A. | N.A. | N.A. | N.A. | N.A. |  |
| A_0018 | 5-Hydroxymethyluracil | 141.030 | 5.40 | 3.5E-04 | N.A. | 4.6E-04 | N.A. | 0.8 | N.A. |  |
| A_0012 | 5-Oxoproline | 128.036 | 9.44 | 4.1E-04 | 9.8E-05 | 3.0E-04 | N.A. | 1.4 | N.A. |  |
| C_0060 | 6-Aminohexanoic acid | 132.102 | 8.07 | 1.1E-04 | 2.1E-05 | N.A. | N.A. | 1< | N.A. |  |
| A_0051 | 6-Phosphogluconic acid | 275.018 | 14.51 | 8.6E-04 | 1.9E-04 | 5.9E-04 | 2.1E-06 | 1.5 | 0.037 | * |
| C_0158 | 8-Hydroxy-2'-deoxyguanosine | 284.099 | 23.04 | N.A. | N.A. | N.A. | N.A. | N.A. | N.A. |  |
| C_0017 | Acetoacetamide | 102.056 | 11.44 | 7.2E-04 | 7.0E-04 | N.A. | N.A. | 1< | N.A. |  |
| C_0070 | Adenine | 136.063 | 7.40 | 6.8E-05 | N.A. | N.A. | N.A. | 1< | N.A. |  |
| C_0151 | Adenosine | 268.104 | 9.66 | 2.5E-05 | N.A. | 3.5E-05 | 1.8E-05 | 0.7 | N.A. |  |
| C_0118 | ADMA | 203.150 | 7.46 | N.A. | N.A. | N.A. | N.A. | N.A. | N.A. |  |
| A_0071 | ADP | 426.023 | 10.83 | 1.1E-02 | 1.7E-03 | 1.2E-02 | 8.1E-04 | 1.0 | 0.793 |  |
| C_0058 | Agmatine | 131.130 | 4.99 | 5.1E-05 | N.A. | 2.9E-05 | 1.5E-06 | 1.8 | N.A. |  |
| C_0012 | Ala | 90.055 | 8.81 | 1.1E-01 | 1.9E-02 | 7.8E-02 | 2.6E-02 | 1.5 | 0.003 | ** |
| C_0039 | *allo*-Threonine | 120.066 | 10.70 | 9.3E-05 | 1.9E-05 | 6.5E-05 | 1.4E-05 | 1.4 | 0.245 |  |
| C_0004 | Aminoacetone | 74.061 | 6.65 | 9.2E-04 | 2.5E-04 | 7.0E-04 | 2.0E-04 | 1.3 | 0.048 | * |
| A_0061 | AMP | 346.057 | 9.22 | 1.0E-03 | 7.1E-04 | 2.0E-03 | 3.3E-03 | 0.5 | 0.385 |  |
| C_0042 | Anserine_divalent | 122.072 | 6.59 | 3.4E-03 | 7.7E-04 | 2.5E-03 | 5.0E-04 | 1.4 | 0.005 | ** |
| C_0104 | Arg | 175.120 | 6.86 | 9.7E-03 | 4.6E-03 | 9.2E-03 | 2.8E-03 | 1.1 | 0.778 |  |
| C_0163 | Argininosuccinic acid | 291.131 | 9.23 | 6.3E-05 | 1.4E-05 | 6.0E-05 | 2.5E-05 | 1.1 | 0.719 |  |
| A_0031 | Ascorbic acid | 175.025 | 8.44 | 5.3E-04 | 2.0E-04 | 6.6E-04 | 4.7E-04 | 0.8 | 0.528 |  |
| C_0064 | Asn | 133.062 | 10.30 | 9.3E-03 | 5.7E-03 | 4.5E-03 | 3.9E-03 | 2.1 | 0.042 | * |
| C_0067 | Asp | 134.045 | 11.41 | 2.9E-03 | 1.2E-03 | 2.8E-03 | 2.2E-03 | 1.1 | 0.833 |  |
| A_0081 | ATP | 505.989 | 11.68 | 1.2E-03 | 7.9E-04 | 9.9E-04 | 4.0E-04 | 1.2 | 0.619 |  |
| C_0034 | Betaine | 118.087 | 11.11 | 6.5E-03 | 3.9E-03 | 2.5E-03 | 4.1E-04 | 2.6 | 0.009 | ** |
| C_0040 | Betaine aldehyde_+H_2_O | 120.103 | 7.24 | 1.3E-04 | 4.8E-05 | 8.0E-05 | 3.7E-05 | 1.6 | 0.016 | * |
| C_0092 | Betonicine | 160.098 | 13.02 | 1.5E-04 | 7.7E-05 | 1.2E-04 | 4.3E-05 | 1.3 | 0.394 |  |
| C_0133 | Butyrylcarnitine | 232.156 | 9.33 | 7.7E-04 | 1.3E-04 | 4.6E-04 | 2.1E-04 | 1.7 | 0.001 | ** |
| C_0018 | Cadaverine | 103.123 | 4.80 | N.A. | N.A. | N.A. | N.A. | N.A. | N.A. |  |
| C_0124 | Carboxymethyllysine | 205.120 | 9.01 | 2.4E-04 | 1.4E-04 | 1.3E-04 | 4.6E-05 | 1.9 | 0.031 | * |
| C_0095 | Carnitine | 162.114 | 8.28 | 1.9E-02 | 5.7E-03 | 1.7E-02 | 4.5E-03 | 1.2 | 0.229 |  |
| C_0129 | Carnosine | 227.115 | 6.54 | 9.7E-04 | 1.2E-03 | 1.8E-04 | 5.7E-05 | 5.5 | 0.060 |  |
| A_0079 | CDP-choline | 487.102 | 6.84 | N.A. | N.A. | N.A. | N.A. | N.A. | N.A. |  |
| C_0024 | Choline | 104.107 | 6.59 | 3.1E-02 | 6.0E-03 | 2.7E-02 | 4.3E-03 | 1.1 | 0.171 |  |
| A_0035 | Citric acid | 191.020 | 25.98 | 1.7E-03 | 4.6E-04 | 1.6E-03 | 4.3E-04 | 1.0 | 0.744 |  |
| C_0105 | Citrulline | 176.104 | 10.89 | 3.4E-03 | 2.0E-03 | 1.0E-03 | 2.2E-04 | 3.4 | 0.005 | ** |
| A_0056 | CMP | 322.046 | 9.57 | 1.9E-04 | 5.7E-05 | 2.9E-04 | 4.9E-05 | 0.7 | 0.014 | * |
| C_0065 | Creatine | 133.080 | 8.40 | 1.2E-01 | 9.8E-03 | 1.2E-01 | 8.1E-03 | 1.0 | 0.810 |  |
| C_0030 | Creatinine | 114.067 | 6.96 | 5.6E-03 | 2.1E-03 | 2.3E-03 | 4.4E-04 | 2.4 | 6.5E-04 | *** |
| C_0041 | Cys | 122.028 | 11.15 | 2.0E-04 | 6.2E-05 | N.A. | N.A. | 1< | N.A. |  |
| C_0128 | Cystathionine | 223.076 | 9.72 | 2.0E-03 | 1.2E-03 | 1.4E-03 | 4.4E-04 | 1.5 | 0.144 |  |
| C_0176 | Cysteine glutathione disulfide | 427.097 | 11.50 | 2.3E-04 | 1.9E-04 | 2.5E-04 | 1.9E-04 | 0.9 | 0.880 |  |
| C_0136 | Cystine | 241.033 | 10.83 | 8.2E-05 | 6.3E-05 | 7.2E-05 | 7.7E-05 | 1.1 | 0.776 |  |
| C_0139 | Cytidine | 244.094 | 9.44 | 3.1E-04 | 7.1E-05 | 3.4E-04 | 7.7E-05 | 0.9 | 0.402 |  |
| C_0026 | Diethanolamine | 106.087 | 7.43 | 4.4E-04 | 2.6E-04 | 4.1E-04 | 4.0E-04 | 1.1 | 0.858 |  |
| C_0014 | Dimethylaminoethanol | 90.092 | 6.67 | 1.4E-04 | 5.6E-05 | 8.4E-05 | 7.4E-06 | 1.7 | 0.015 | * |
| C_0148 | Dyphylline | 255.110 | 22.97 | 3.4E-04 | 4.9E-05 | 3.0E-04 | 5.9E-05 | 1.1 | 0.154 |  |
| C_0076 | Ectoine | 143.081 | 9.09 | 1.0E-04 | N.A. | 1.0E-04 | 3.2E-05 | 1.0 | N.A. |  |
| C_0131 | Ergothioneine | 230.097 | 17.64 | N.A. | N.A. | N.A. | N.A. | N.A. | N.A. |  |
| C_0003 | Ethanolamine | 62.061 | 6.13 | 3.5E-03 | 8.0E-04 | 2.4E-03 | 3.7E-04 | 1.4 | 0.003 | ** |
| A_0017 | Ethanolamine phosphate | 140.012 | 7.99 | 9.6E-04 | 5.8E-04 | 4.7E-04 | 1.4E-04 | 2.0 | 0.027 | * |
| A_0059 | Fructose 1,6-diphosphate | 338.990 | 14.45 | 4.0E-04 | N.A. | N.A. | N.A. | 1< | N.A. |  |
| A_0050 | Fructose 6-phosphate | 259.024 | 9.90 | 4.4E-03 | 6.4E-03 | 3.3E-03 | 4.1E-03 | 1.3 | 0.668 |  |
| A_0008 | Fumaric acid | 115.004 | 24.92 | 1.0E-03 | 2.7E-04 | 8.4E-04 | N.A. | 1.2 | N.A. |  |
| C_0020 | GABA | 104.071 | 7.46 | 2.0E-03 | 2.0E-03 | 7.5E-04 | 2.1E-04 | 2.6 | 0.093 |  |
| C_0107 | Galactosamine Glucosamine | 180.088 | 8.97 | 9.4E-05 | 3.9E-05 | 9.0E-05 | 2.5E-05 | 1.1 | 0.762 |  |
| A_0073 | GDP | 442.019 | 10.60 | 1.1E-03 | 1.8E-04 | 9.9E-04 | 9.4E-05 | 1.1 | 0.040 | * |
| C_0081 | Gln | 147.077 | 10.58 | 6.1E-02 | 1.9E-02 | 4.1E-02 | 1.5E-02 | 1.5 | 0.016 | * |
| C_0084 | Glu | 148.061 | 10.78 | 5.0E-02 | 1.2E-02 | 2.9E-02 | 8.5E-03 | 1.7 | 2.7E-04 | *** |
| C_0155 | Glu-Glu | 277.105 | 10.74 | 2.4E-05 | 1.8E-06 | 3.2E-05 | 7.4E-06 | 0.8 | 0.084 |  |
| A_0039 | Gluconic acid | 195.051 | 8.05 | 3.8E-04 | N.A. | N.A. | N.A. | 1< | N.A. |  |
| C_0106 | Gluconolactone | 179.055 | 23.73 | N.A. | N.A. | N.A. | N.A. | N.A. | N.A. |  |
| A_0048 | Glucose 1-phosphate | 259.024 | 10.07 | 1.6E-03 | 1.7E-03 | 1.7E-03 | 7.1E-04 | 0.9 | 0.904 |  |
| A_0049 | Glucose 6-phosphate | 259.024 | 9.77 | 1.5E-02 | 2.3E-02 | 1.1E-02 | 1.6E-02 | 1.4 | 0.640 |  |
| C_0167 | Glutathione (GSH) | 308.093 | 13.15 | 5.2E-03 | 3.1E-03 | 1.4E-03 | N.A. | 3.8 | N.A. |  |
| C_0166 | Glutathione (GSSG)_divalent | 307.085 | 12.05 | 2.9E-03 | 1.6E-03 | 2.9E-03 | 1.0E-03 | 1.0 | 0.994 |  |
| C_0007 | Gly | 76.040 | 8.10 | 8.4E-02 | 2.2E-02 | 5.6E-02 | 8.7E-03 | 1.5 | 0.003 | ** |
| C_0114 | Gly-Asp | 191.068 | 9.69 | 1.5E-04 | 2.8E-05 | 9.5E-05 | 2.7E-05 | 1.5 | 5.5E-04 | *** |
| C_0063 | Gly-Gly | 133.061 | 8.16 | 2.6E-04 | 5.3E-05 | 2.5E-04 | 6.3E-05 | 1.0 | 0.723 |  |
| C_0015 | Glycerol | 93.055 | 22.89 | 5.4E-02 | 1.0E-02 | 5.6E-02 | 1.4E-02 | 1.0 | 0.765 |  |
| A_0028 | Glycerol 3-phosphate | 171.007 | 12.00 | 2.8E-02 | 2.8E-03 | 2.6E-02 | 4.5E-03 | 1.1 | 0.310 |  |
| C_0149 | Glycerophosphocholine | 258.112 | 22.41 | 2.4E-03 | 1.0E-03 | 1.4E-03 | 1.6E-04 | 1.7 | 0.010 | ** |
| A_0063 | GMP | 362.052 | 9.11 | 2.1E-04 | N.A. | N.A. | N.A. | 1< | N.A. |  |
| C_0089 | Guanine | 152.061 | 8.09 | 5.1E-04 | 2.8E-04 | 2.5E-04 | 1.2E-04 | 2.0 | 0.021 | * |
| C_0159 | Guanosine | 284.101 | 12.41 | 8.3E-04 | 1.8E-04 | 8.7E-04 | 2.2E-04 | 0.9 | 0.597 |  |
| C_0160 | His-Glu | 285.121 | 7.26 | N.A. | N.A. | N.A. | N.A. | N.A. | N.A. |  |
| C_0091 | His | 157.080 | 6.97 | 1.6E-03 | 3.7E-04 | 1.0E-03 | 2.4E-04 | 1.5 | 0.001 | ** |
| C_0137 | Homocarnosine | 241.131 | 6.59 | 6.9E-02 | 1.7E-02 | 4.7E-02 | 1.1E-02 | 1.5 | 0.004 | ** |
| C_0113 | Homocitrulline | 190.120 | 11.01 | 9.2E-05 | 3.8E-05 | 6.0E-05 | N.A. | 1.5 | N.A. |  |
| C_0059 | Hydroxyproline | 132.067 | 11.90 | 1.3E-03 | 1.2E-04 | 1.2E-03 | 3.5E-04 | 1.0 | 0.782 |  |
| C_0027 | Hypotaurine | 110.028 | 18.45 | 6.0E-04 | 6.3E-04 | 1.2E-04 | 3.2E-05 | 5.0 | 0.038 | * |
| C_0071 | Hypoxanthine | 137.047 | 10.96 | 9.6E-04 | 4.3E-04 | 7.4E-04 | 2.7E-04 | 1.3 | 0.194 |  |
| A_0072 | IDP | 427.007 | 11.04 | 9.4E-04 | 6.2E-04 | 2.6E-03 | 2.4E-03 | 0.4 | 0.063 |  |
| C_0061 | Ile | 132.102 | 10.03 | 7.0E-02 | 1.1E-02 | 4.9E-02 | 1.4E-02 | 1.4 | 0.002 | ** |
| C_0050 | Imidazole-4-acetic acid | 127.050 | 7.82 | N.A. | N.A. | 3.0E-05 | 1.0E-05 | <1 | N.A. |  |
| A_0062 | IMP | 347.041 | 9.55 | 1.8E-01 | 2.6E-02 | 2.1E-01 | 1.3E-02 | 0.8 | 0.002 | ** |
| C_0153 | Inosine | 269.089 | 20.00 | 4.2E-03 | 8.1E-04 | 8.9E-03 | 6.8E-03 | 0.5 | 0.059 |  |
| A_0011 | Isethionic acid | 124.992 | 11.52 | 3.3E-04 | 7.2E-05 | 3.6E-04 | 3.8E-05 | 0.9 | 0.374 |  |
| C_0006 | Isobutylamine | 74.097 | 6.79 | 3.9E-04 | 5.3E-06 | 4.1E-04 | 1.2E-04 | 1.0 | 0.803 |  |
| C_0132 | Isobutyrylcarnitine | 232.156 | 9.23 | 1.7E-04 | 9.1E-05 | 1.6E-04 | 9.3E-05 | 1.1 | 0.824 |  |
| C_0045 | Isonicotinamide | 123.056 | 7.05 | N.A. | N.A. | N.A. | N.A. | N.A. | N.A. |  |
| C_0141 | Isovalerylcarnitine | 246.169 | 9.57 | 1.5E-04 | 2.4E-05 | 2.0E-04 | 1.1E-04 | 0.7 | 0.356 |  |
| A_0005 | Lactic acid | 89.025 | 10.78 | 1.1E+00 | 1.5E-01 | 1.1E+00 | 1.4E-01 | 1.0 | 0.922 |  |
| A_0041 | Lauric acid | 199.171 | 7.65 | 3.0E-04 | N.A. | 3.9E-04 | N.A. | 0.8 | N.A. |  |
| C_0173 | Lauroylcarnitine | 344.282 | 10.93 | 8.2E-05 | 5.2E-05 | 7.4E-05 | 3.4E-05 | 1.1 | 0.711 |  |
| C_0062 | Leu | 132.102 | 10.13 | 9.9E-02 | 1.5E-02 | 7.8E-02 | 2.2E-02 | 1.3 | 0.024 | * |
| C_0082 | Lys | 147.113 | 6.65 | 3.8E-02 | 1.3E-02 | 2.7E-02 | 9.1E-03 | 1.4 | 0.049 | * |
| A_0014 | Malic acid | 133.015 | 21.07 | 1.1E-02 | 3.2E-03 | 6.9E-03 | 3.0E-03 | 1.6 | 0.013 | * |
| C_0086 | Met | 150.059 | 10.55 | 1.5E-02 | 5.2E-03 | 1.6E-02 | 4.5E-03 | 1.0 | 0.795 |  |
| C_0097 | Methionine sulfoxide | 166.053 | 11.62 | 2.5E-04 | 1.1E-04 | 2.6E-04 | 2.2E-04 | 1.0 | 0.922 |  |
| C_0125 | Methoxamine | 212.129 | 9.08 | 4.3E-05 | 4.4E-06 | N.A. | N.A. | 1< | N.A. |  |
| C_0005 | Methylguanidine | 74.073 | 5.97 | 3.5E-04 | N.A. | N.A. | N.A. | 1< | N.A. |  |
| C_0101 | Metronidazole | 172.073 | 9.26 | N.A. | N.A. | N.A. | N.A. | N.A. | N.A. |  |
| A_0047 | *myo*-Inositol 2-phosphate | 259.023 | 10.44 | 6.1E-04 | 1.3E-04 | 6.7E-04 | 2.8E-04 | 0.9 | 0.611 |  |
| C_0023 | *N*,*N*-Dimethylglycine | 104.071 | 10.76 | 4.5E-04 | 1.6E-04 | 9.0E-05 | 2.9E-05 | 5.0 | 4.4E-05 | *** |
| A_0030 | *N*-Acetylaspartic acid | 174.042 | 14.44 | 4.4E-04 | 1.1E-04 | 6.0E-04 | N.A. | 0.7 | N.A. |  |
| C_0116 | *N*-Acetylhistidine | 198.089 | 9.62 | 5.9E-05 | 4.3E-05 | N.A. | N.A. | 1< | N.A. |  |
| C_0110 | *N*-Acetyllysine | 189.123 | 9.57 | 3.2E-04 | 1.8E-04 | 2.4E-04 | 7.4E-05 | 1.3 | 0.250 |  |
| A_0053 | *N*-Acetylneuraminic acid | 308.101 | 7.22 | N.A. | N.A. | N.A. | N.A. | N.A. | N.A. |  |
| C_0103 | *N*-Acetylornithine | 175.109 | 9.35 | 1.5E-04 | 4.6E-05 | N.A. | N.A. | 1< | N.A. |  |
| C_0085 | *N*-Acetylserine | 148.062 | 24.10 | N.A. | N.A. | N.A. | N.A. | N.A. | N.A. |  |
| C_0056 | *N*-Methylproline | 130.087 | 12.34 | N.A. | N.A. | N.A. | N.A. | N.A. | N.A. |  |
| C_0068 | *N*-Nitrosodiethanolamine | 135.076 | 22.87 | N.A. | N.A. | N.A. | N.A. | N.A. | N.A. |  |
| C_0102 | *N*^5^-Ethylglutamine | 175.109 | 11.21 | N.A. | N.A. | N.A. | N.A. | N.A. | N.A. |  |
| C_0112 | *N*^6^,*N*^6^,*N*^6^-Trimethyllysine | 189.161 | 6.90 | 6.2E-04 | 3.4E-04 | 5.2E-04 | 1.9E-04 | 1.2 | 0.472 |  |
| C_0111 | *N*^6^-Acetyllysine | 189.124 | 11.29 | N.A. | N.A. | N.A. | N.A. | N.A. | N.A. |  |
| C_0093 | *N*^6^-Methyllysine | 161.130 | 6.85 | 5.8E-05 | 2.0E-05 | 6.6E-05 | 2.1E-05 | 0.9 | 0.409 |  |
| A_0086 | NAD^+^ | 662.102 | 6.61 | 3.7E-03 | 7.8E-04 | 3.7E-03 | 5.3E-04 | 1.0 | 0.795 |  |
| C_0046 | Nicotinamide | 123.056 | 7.20 | 6.5E-03 | 1.9E-03 | 4.8E-03 | 1.4E-03 | 1.3 | 0.039 | * |
| C_0047 | Nicotinic acid | 124.040 | 9.81 | 4.7E-05 | 1.5E-05 | 7.5E-05 | 1.3E-05 | 0.6 | 0.001 | ** |
| C_0172 | NMN | 335.066 | 21.29 | 7.3E-05 | 2.3E-05 | 9.0E-05 | 3.6E-05 | 0.8 | 0.403 |  |
| C_0121 | *O*-Acetylcarnitine | 204.124 | 8.84 | 1.1E-03 | 4.7E-04 | 9.0E-04 | 4.9E-04 | 1.2 | 0.463 |  |
| C_0094 | *O*-Acetylhomoserine 2-Aminoadipic acid | 162.077 | 10.80 | 1.3E-04 | 8.1E-05 | 1.0E-04 | 1.8E-05 | 1.3 | 0.370 |  |
| C_0161 | Octanoylcarnitine | 288.219 | 10.21 | 1.0E-04 | 4.1E-05 | 9.5E-05 | 2.0E-05 | 1.1 | 0.654 |  |
| C_0143 | Octopine | 247.138 | 10.55 | 7.0E-05 | 3.3E-05 | 7.6E-05 | 2.8E-05 | 0.9 | 0.785 |  |
| C_0162 | Ophthalmic acid | 290.136 | 13.13 | 5.3E-05 | 2.7E-05 | N.A. | N.A. | 1< | N.A. |  |
| C_0066 | Ornithine | 133.098 | 6.58 | 5.3E-04 | 2.4E-04 | 1.8E-04 | 4.8E-05 | 3.0 | 0.001 | ** |
| A_0016 | *p*-Toluic acid *m*-Toluic acid *o*-Toluic acid | 135.046 | 9.10 | 9.3E-04 | 2.1E-04 | 9.7E-04 | 3.6E-04 | 1.0 | 0.765 |  |
| A_0042 | Pantothenic acid | 218.104 | 7.66 | 2.1E-03 | 1.4E-03 | 9.2E-04 | 3.2E-04 | 2.3 | 0.020 | * |
| A_0022 | Pelargonic acid | 157.124 | 8.11 | 4.3E-04 | 2.2E-05 | 3.4E-04 | 7.3E-05 | 1.2 | 0.049 | * |
| C_0098 | Phe | 166.087 | 10.92 | 3.1E-02 | 7.3E-03 | 2.3E-02 | 5.5E-03 | 1.3 | 0.014 | * |
| C_0109 | Phosphorylcholine | 184.074 | 21.16 | 1.9E-03 | 4.8E-04 | 1.6E-03 | 2.7E-04 | 1.2 | 0.122 |  |
| C_0054 | Pipecolic acid | 130.087 | 10.09 | 9.4E-04 | 7.3E-04 | 4.1E-04 | 1.7E-04 | 2.3 | 0.049 | * |
| C_0031 | Pro | 116.071 | 10.64 | 3.4E-02 | 7.3E-03 | 2.3E-02 | 6.3E-03 | 1.5 | 0.002 | ** |
| A_0002 | Propionic acid | 73.030 | 10.86 | N.A. | N.A. | N.A. | N.A. | N.A. | N.A. |  |
| C_0126 | Propionylcarnitine XC0061 | 218.140 | 9.02 | 2.3E-03 | 7.4E-04 | 7.2E-04 | 2.0E-04 | 3.2 | 5.4E-05 | *** |
| A_0065 | PRPP | 388.946 | 16.48 | 5.5E-04 | 1.7E-04 | 3.7E-04 | 5.1E-05 | 1.5 | 0.034 | * |
| C_0010 | Putrescine | 89.108 | 4.53 | 1.7E-04 | 1.1E-04 | 1.1E-04 | 2.9E-05 | 1.5 | 0.135 |  |
| C_0144 | Pyridoxamine 5'-phosphate | 249.065 | 10.29 | 5.3E-05 | 1.1E-05 | 4.7E-05 | 1.1E-05 | 1.1 | 0.232 |  |
| A_0032 | Pyrophosphate | 176.937 | 16.54 | 8.5E-03 | N.A. | 7.2E-03 | 4.0E-04 | 1.2 | N.A. |  |
| A_0004 | Pyruvic acid | 87.009 | 12.78 | 5.6E-03 | N.A. | N.A. | N.A. | 1< | N.A. |  |
| A_0038 | Quinic acid | 191.058 | 8.08 | 2.3E-04 | 1.8E-05 | N.A. | N.A. | 1< | N.A. |  |
| A_0043 | Ribose 5-phosphate | 229.013 | 10.49 | 3.2E-04 | N.A. | N.A. | N.A. | 1< | N.A. |  |
| A_0054 | Ribulose 1,5-diphosphate | 308.979 | 15.52 | N.A. | N.A. | N.A. | N.A. | N.A. | N.A. |  |
| A_0044 | Ribulose 5-phosphate | 229.013 | 10.88 | 9.3E-04 | 3.5E-04 | 5.6E-04 | 1.5E-04 | 1.7 | 0.010 | ** |
| C_0174 | *S*-Adenosylhomocysteine | 385.131 | 8.38 | 2.3E-05 | 6.0E-06 | 2.6E-05 | 5.9E-06 | 0.9 | 0.529 |  |
| C_0175 | *S*-Adenosylmethionine | 399.146 | 6.86 | 3.7E-04 | 1.4E-04 | 3.4E-04 | 6.7E-05 | 1.1 | 0.563 |  |
| C_0069 | *S*-Methylcysteine | 136.043 | 11.41 | 8.4E-05 | 6.6E-06 | 6.6E-05 | 2.7E-05 | 1.3 | 0.512 |  |
| C_0170 | *S*-Methylglutathione | 322.108 | 13.29 | 2.6E-05 | 8.7E-06 | N.A. | N.A. | 1< | N.A. |  |
| C_0156 | Saccharopine | 277.142 | 10.56 | 1.4E-04 | 7.6E-05 | 1.8E-04 | 1.4E-04 | 0.8 | 0.422 |  |
| C_0011 | Sarcosine | 90.055 | 9.28 | 4.9E-04 | 1.4E-04 | 3.0E-04 | 6.9E-05 | 1.6 | 0.002 | ** |
| C_0119 | SDMA | 203.151 | 7.59 | 4.6E-05 | 1.3E-05 | 3.0E-05 | N.A. | 1.5 | N.A. |  |
| A_0052 | Sedoheptulose 7-phosphate | 289.034 | 9.58 | 1.6E-04 | N.A. | N.A. | N.A. | 1< | N.A. |  |
| C_0025 | Ser | 106.050 | 9.79 | 2.3E-02 | 5.6E-03 | 1.7E-02 | 4.1E-03 | 1.4 | 0.007 | ** |
| C_0135 | Ser-Glu | 235.093 | 10.21 | 2.9E-05 | 2.1E-06 | 3.1E-05 | 5.6E-06 | 0.9 | 0.441 |  |
| C_0080 | Spermidine | 146.166 | 4.36 | 3.3E-04 | 1.2E-04 | 3.6E-04 | 8.1E-05 | 0.9 | 0.470 |  |
| C_0120 | Spermine | 203.224 | 4.30 | 3.7E-05 | N.A. | N.A. | N.A. | 1< | N.A. |  |
| C_0077 | Stachydrine | 144.102 | 11.36 | 8.3E-05 | 3.9E-05 | 3.9E-05 | N.A. | 2.1 | N.A. |  |
| A_0010 | Succinic acid | 117.020 | 20.69 | 5.4E-03 | 1.5E-03 | 4.2E-03 | 1.8E-03 | 1.3 | 0.146 |  |
| C_0048 | Taurine | 126.022 | 22.88 | 1.1E-01 | 7.6E-03 | 1.1E-01 | 1.2E-02 | 1.0 | 0.748 |  |
| A_0082 | Taurocholic acid | 514.284 | 6.76 | N.A. | N.A. | N.A. | N.A. | N.A. | N.A. |  |
| A_0023 | Terephthalic acid | 165.019 | 16.34 | 9.8E-04 | 1.0E-04 | 9.4E-04 | 9.7E-05 | 1.1 | 0.301 |  |
| C_0145 | Tetrahydrouridine | 249.106 | 22.91 | N.A. | N.A. | N.A. | N.A. | N.A. | N.A. |  |
| C_0037 | Thr | 120.066 | 10.33 | 2.6E-02 | 5.8E-03 | 2.2E-02 | 4.6E-03 | 1.2 | 0.103 |  |
| C_0083 | *threo*-β-Methylaspartic acid | 148.061 | 12.19 | 6.5E-05 | N.A. | 9.5E-05 | 2.0E-05 | 0.7 | N.A. |  |
| A_0015 | Threonic acid | 135.030 | 9.19 | N.A. | N.A. | N.A. | N.A. | N.A. | N.A. |  |
| C_0057 | *trans*-Glutaconic acid | 131.035 | 23.85 | 3.8E-04 | 7.4E-05 | 2.3E-04 | N.A. | 1.6 | N.A. |  |
| C_0088 | Triethanolamine | 150.114 | 8.03 | 7.5E-05 | 3.0E-05 | 7.6E-05 | 4.8E-05 | 1.0 | 0.957 |  |
| C_0072 | Trigonelline | 138.055 | 10.28 | 2.5E-04 | 1.6E-04 | 2.0E-04 | 1.2E-04 | 1.2 | 0.627 |  |
| C_0001 | Trimethylamine | 60.081 | 5.65 | 2.6E-04 | 1.9E-04 | 1.5E-04 | 5.4E-05 | 1.7 | 0.342 |  |
| C_0008 | Trimethylamine *N*-oxide | 77.079 | 6.27 | 1.1E-02 | 3.5E-03 | 2.0E-02 | 1.8E-03 | 0.6 | 8.7E-06 | *** |
| C_0123 | Trp | 205.098 | 10.85 | 5.3E-03 | 1.7E-03 | 5.1E-03 | 1.4E-03 | 1.0 | 0.752 |  |
| C_0108 | Tyr | 182.082 | 11.20 | 1.3E-02 | 4.7E-03 | 1.3E-02 | 3.1E-03 | 1.0 | 0.759 |  |
| C_0099 | Tyr-Arg_divalent | 169.596 | 7.53 | N.A. | N.A. | N.A. | N.A. | N.A. | N.A. |  |
| C_0074 | Tyramine | 138.094 | 8.07 | 4.2E-05 | N.A. | N.A. | N.A. | 1< | N.A. |  |
| A_0068 | UDP | 402.996 | 11.56 | 7.3E-04 | 1.8E-04 | 6.6E-04 | 1.4E-04 | 1.1 | 0.370 |  |
| A_0084 | UDP-glucose UDP-galactose | 565.050 | 8.59 | 7.2E-04 | 2.3E-04 | 6.9E-04 | 2.4E-04 | 1.0 | 0.783 |  |
| A_0085 | UDP-*N*-acetylgalactosamine UDP-*N*-acetylglucosamine | 606.074 | 8.41 | 5.2E-04 | N.A. | 2.1E-04 | 4.9E-06 | 2.5 | N.A. |  |
| A_0057 | UMP | 323.030 | 9.73 | 4.6E-04 | 1.3E-04 | 4.2E-04 | 9.3E-05 | 1.1 | 0.393 |  |
| C_0029 | Uracil | 113.035 | 22.88 | N.A. | N.A. | N.A. | N.A. | N.A. | N.A. |  |
| C_0002 | Urea | 61.040 | 21.82 | 4.6E-02 | 1.5E-02 | 3.6E-02 | 8.3E-03 | 1.3 | 0.118 |  |
| A_0025 | Uric acid | 167.021 | 8.87 | N.A. | N.A. | N.A. | N.A. | N.A. | N.A. |  |
| C_0140 | Uridine | 245.081 | 23.02 | 1.2E-04 | 2.8E-05 | N.A. | N.A. | 1< | N.A. |  |
| C_0033 | Val | 118.087 | 9.83 | 6.6E-02 | 9.4E-03 | 4.9E-02 | 1.2E-02 | 1.3 | 0.003 | ** |
| A_0036 | XA0019 | 191.021 | 8.27 | N.A. | N.A. | 3.3E-04 | 1.1E-04 | <1 | N.A. |  |
| A_0045 | XA0033 | 242.080 | 7.43 | N.A. | N.A. | N.A. | N.A. | N.A. | N.A. |  |
| A_0074 | XA0065 | 445.054 | 6.98 | 2.4E-04 | 2.6E-05 | N.A. | N.A. | 1< | N.A. |  |
| C_0090 | Xanthine | 153.042 | 19.92 | N.A. | N.A. | N.A. | N.A. | N.A. | N.A. |  |
| C_0052 | XC0016 | 129.067 | 8.36 | 9.0E-05 | 3.2E-05 | 1.7E-04 | 1.3E-04 | 0.5 | 0.103 |  |
| C_0078 | XC0029 | 144.102 | 12.11 | 5.8E-05 | N.A. | 5.3E-05 | N.A. | 1.1 | N.A. |  |
| C_0134 | XC0071 | 234.182 | 6.01 | 4.5E-05 | 7.3E-06 | 4.5E-05 | 6.3E-06 | 1.0 | 0.964 |  |
| C_0147 | XC0089 Nicotinamide riboside | 255.099 | 9.32 | N.A. | N.A. | N.A. | N.A. | N.A. | N.A. |  |
| C_0168 | XC0126 | 310.114 | 14.96 | N.A. | N.A. | N.A. | N.A. | N.A. | N.A. |  |
| C_0171 | XC0132 | 325.162 | 8.37 | 2.6E-05 | 4.1E-06 | 3.0E-05 | 9.3E-06 | 0.9 | 0.435 |  |
| C_0013 | β-Ala | 90.056 | 7.13 | 5.9E-04 | 1.5E-04 | 4.9E-04 | 1.3E-04 | 1.2 | 0.139 |  |
| C_0079 | γ-Butyrobetaine | 146.118 | 7.88 | 8.0E-03 | 1.8E-03 | 5.7E-03 | 1.4E-03 | 1.4 | 0.005 | ** |
| C_0127 | γ-Glu-Ala | 219.098 | 12.18 | 1.8E-04 | 8.0E-05 | 6.7E-05 | 3.8E-05 | 2.7 | 0.001 | ** |
| C_0154 | γ-Glu-Gln | 276.120 | 12.90 | 1.1E-04 | 4.2E-05 | 6.6E-05 | 1.9E-05 | 1.7 | 0.027 | * |
| C_0122 | γ-Glu-Gly | 205.084 | 11.91 | 1.5E-04 | 4.9E-05 | 4.9E-05 | 2.2E-05 | 3.1 | 1.0E-04 | *** |
| C_0150 | γ-Glu-Ile γ-Glu-Leu | 261.146 | 12.81 | 3.1E-04 | 1.2E-04 | 1.3E-04 | 5.9E-05 | 2.4 | 8.5E-04 | *** |
| C_0075 | γ-Glu-Lys_divalent | 138.582 | 8.37 | N.A. | N.A. | N.A. | N.A. | N.A. | N.A. |  |
| C_0157 | γ-Glu-Met | 279.103 | 12.85 | 1.4E-04 | 4.7E-05 | 7.8E-05 | 4.7E-05 | 1.9 | 0.008 | ** |
| C_0164 | γ-Glu-Phe | 295.127 | 12.93 | 6.3E-05 | 6.6E-06 | N.A. | N.A. | 1< | N.A. |  |
| A_0046 | γ-Glu-Taurine | 253.051 | 8.10 | 2.3E-04 | 2.7E-05 | N.A. | N.A. | 1< | N.A. |  |
| C_0146 | γ-Glu-Thr | 249.110 | 12.61 | N.A. | N.A. | N.A. | N.A. | N.A. | N.A. |  |
| C_0169 | γ-Glu-Tyr | 311.127 | 13.13 | N.A. | N.A. | N.A. | N.A. | N.A. | N.A. |  |
| C_0142 | γ-Glu-Val | 247.130 | 12.59 | 4.9E-05 | 5.8E-06 | N.A. | N.A. | 1< | N.A. |  |

ID consists of analysis mode and number. 'C' and 'A' showed cation and anion modes, respectively

N.D. (Not Detected): The metabolite was below the detection limits.

N.A. (Not Available): The calculation was not possible.

^¶^ In the ratio calculation, the latter was denominator.

^||^ The p-value in Welch's t-test. * < 0.05, ** < 0.01, *** < 0.001

They were sorted by the ratio of treatment to control in descending order.
